# Supplementary material for: Splice-Junction-Based Mapping of Alternative Isoforms in the Human Proteome
Source: Cell Rep. Author manuscript; Available in PMC 2020 Jan 15. (PMC6961840; doi:10.1016/j.celrep.2019.11.026)
Supplement: 2 [file NIHMS1546469-supplement-2.zip › DF1/PXD006675/RightVentricle/RightVentricle_51_ACTG1_VPHIDNGSGMCKAGFAGDDAPR.pdf]

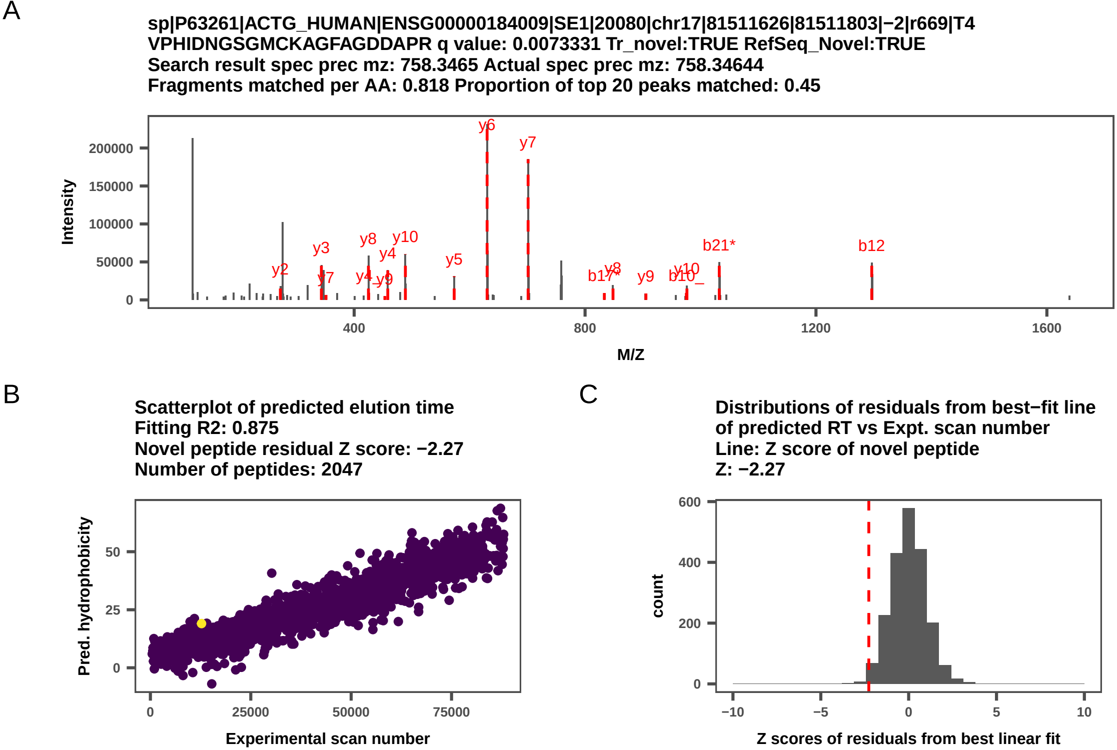

A
sp|P63261|ACTG_HUMAN|ENSG00000184009|SE1|20080|chr17|81511626|81511803|−2|r669|T4
VPHIDNGSGMCKAGFAGDDAPR q value: 0.0073331 Tr_novel:TRUE RefSeq_Novel:TRUE
Search result spec prec mz: 758.3465 Actual spec prec mz: 758.34644
Fragments matched per AA: 0.818 Proportion of top 20 peaks matched: 0.45
Intensity
200000
150000
100000
50000
0
y6
y7
y3
y8
y10
y4
y5
y2
y7
y4
y9
b17*
y8
y9
b10
y10
b21*
b12
400
800
1200
1600
M/Z
B
Scatterplot of predicted elution time
Fitting R2: 0.875
Novel peptide residual Z score: −2.27
Number of peptides: 2047
Pred. hydrophobicity
50
25
0
0
25000
50000
75000
Experimental scan number
C
Distributions of residuals from best−fit line
of predicted RT vs Expt. scan number
Line: Z score of novel peptide
Z: −2.27
count
600
400
200
0
−10
−5
0
5
10
Z scores of residuals from best linear fit
